# Stability Study of Baclofen in an Oral Powder Form Compounded for Pediatric Patients in Japan

**DOI:** 10.3390/children9091313

**Published:** 2022-08-29

**Authors:** Jumpei Saito, Takehisa Hanawa, Ayuna Ozawa, Takahiro Matsumoto, Nozomi Yoshikawa, Tsutomu Harada, Kana Iwahashi, Akimasa Yamatani

**Affiliations:** 1 Department of Pharmacy, National Center for Child Health and Development, Tokyo 157-0054, Japan; 2 Division of Clinical Pharmacology and Oral Formulation Development, National Center for Child Health and Development, Tokyo 157-0054, Japan; 3 Faculty of Pharmaceutical Sciences, Tokyo University of Science, Chiba 278-8510, Japan; 4 R&D Center, Ohara Pharmaceutical Co., Ltd., Koka 520-3403, Japan; 5 Division of Pharmaceutics, School of Pharmacy, Showa University, Tokyo 142-8555, Japan

**Keywords:** baclofen, pediatric formulation, oral powder, stability, quality assurance

## Abstract

Baclofen is used as a skeletal muscle relaxant for multiple sclerosis patients and pediatric patients with cerebral palsy and is prescribed to pediatric patients at 0.3 to 1.0 mg/kg/dose. Baclofen tablets, an oral drug, are usually administered as a powder in pediatric wards after a formulation change by the pharmacist. However, there is no information about stability and assurance of quality for compounded products. The purpose of this study was to design a 10 mg/g oral powder of baclofen and to investigate the stability and changes in the physical properties of this compounded product. A 10 mg/g baclofen powder was prepared by adding extra-fine crystal lactose hydrate to crushed and filtrated baclofen tablets and was stored in a polycarbonate amber bottle with desiccant or in a coated paper laminated with cellophane and polyethylene. The stability of baclofen at 25 ± 2 °C/60 ± 5%RH was tested for 120 days in ‘bottle (closed)’, ‘bottle (in use)’, and ‘laminated’ storage conditions. Baclofen concentrations ranged from 90.0% to 110.0% of the initial concentration under all storage conditions. No crystallographic or dissolution changes were observed after storage. This information can help with the management of baclofen compounded powder in pharmacies.

## 1. Introduction

Baclofen is a structural analog of the inhibitory neurotransmitter gamma-aminobutyric acid (GABA) [1] and is an oral therapy for pediatric patients with cerebral palsy (CP) [2,3,4,5,6]. The previous observational study in a sample of pediatricians who manage pediatric patients with CP showed that baclofen was the first-line medication to manage dystonia [7,8]. Despite the widespread use of oral baclofen in pediatric patients with CP, in Japan, only tablet form is available for oral baclofen formulation. In previous pharmacokinetic studies, pediatric patients were started at 2.5 mg three times daily, half the adult dose, and slowly titrated up (from 2.5 mg three times daily to a maximum tolerated dose of 20 mg four times daily) until efficacy was observed [9]. The use of oral baclofen has also been shown in neonates with CP or neonatal hypertonia at an initial dose of 0.3 to 0.5 mg/kg/day [10,11]. The liquid form is available outside of Japan. The dosage was set as ‘Treatment should usually be started with a shallow dose (corresponding to approximately 0.3 mg/kg a day), in 2–4 divided doses. The dosage should be raised cautiously until it becomes sufficient for the child’s requirements.’

In these situations, pediatric compounding, such as tablet crushing or dissolving with vehicles for dosage adjustment and improving their swallowability, cannot be avoided. Moreover, in pediatric patients with CP, tablet crushing is forced occasionally because oral baclofen is sometimes administered via an enteral or nasogastric tube [12,13]. Referring to the results of the survey of 208 pediatric hospitals in Japan, approximately 19.2% of the facilities implemented formulation changes in baclofen oral tablets for pediatric patients [14]. In the compounding process of baclofen tablets, lactose hydrate is commonly used in Japan to ensure uniformity of dispensing when the tablets are crushed. Unlike the ‘Standardize 4 Safety initiative’ in the American Society of Health-System Pharmacists [15], there is no information on a standardized method for pediatric compounding for baclofen tablets in Japan. It should be noted that this off-label use is not assured according to the product label.

In addition, because the manufacturer has not provided data on the stability of baclofen tablets, the quality of compounded baclofen tablets cannot be guaranteed for proper drug therapy, and there is a risk that both the safety and efficacy of the drug may be affected. Therefore, to assure the efficacy and safety of pediatric drugs, physicochemical stability tests (stability and dissolution tests) of active pharmaceutical ingredients must be conducted following the ICH (International Council for Harmonization of Technical Requirements for Pharmaceuticals for Human Use) guidelines [16]. Unfortunately, to our knowledge, there is no published stability study on baclofen powder form formulated by crushing commercially available tablets into powders. Furthermore, information on the compatibility of crushed baclofen tablets with lactose hydrate and cornstarch, which are typical diluents in Japanese clinical practice, is extremely important.

Our aims of this study are to formulate an oral powder form of baclofen in extra-fine crystal lactose hydrate (10.0 mg/g) and to assess the stability and physical properties of baclofen in ‘bottle (closed)’, ‘bottle (in use)’, and ‘laminated’ storage conditions.

## 2. Materials and Methods

### 2.1. Reagents and Test Solution Preparation

All reagents and solvents were of analytical grade. Water for chromatographic analysis was purified from reverse osmosis systems (Merk Millipore, Darmstadt, Germany). Baclofen (target substance, Sigma-Aldrich, Tokyo, Japan) and baclofen impurities A and B (Sigma-Aldrich, Tokyo, Japan), the major impurities in baclofen in previous reports [17], were used as standards (purity > 99.0%). Lactose monohydrate (extra-fine crystal lactose hydrate ‘Hoei’, Pfizer Co., Ltd., Tokyo, Japan) was used as a diluent. Standard solutions of baclofen (20.0 µg/mL) and baclofen impurities A and B (20.0 µg/mL) were prepared by dissolving 2 mg of each substance in 100 mL of a 50% (*v*/*v*) methanol/water mixture. For the test solution, 1.0 g of stored compound powder (10.0 mg baclofen) was dissolved in 100 mL of 50% (*v*/*v*) methanol/water mixture and then diluted with the mixed solvents of the mobile phase of high-performance liquid chromatography (60% (*v*/*v*) 10 mM ammonium formate solution and 0.1% acetonitrile formate solution) to prepare the baclofen solution. The test solution was prepared in triplicate.

### 2.2. Baclofen Powder Compounding

Baclofen powder was prepared in the formulation laboratory within the Department of Pharmacy of the National Centre for Child Health and Development, which conforms to the regulations on pharmacy buildings and equipment [18]. Five hundred 10 mg baclofen tablets (5000 mg as baclofen, total weight of about 65.0 g) (Gabalon, Alfresa Corporation, Osaka, Japan) were crushed by an automatic tablet crusher (KC-HUK2, Konishi Medical Instruments Corporation, Osaka, Japan) at 6000 rpm for 30 s. The crushed tablets were filtered through a Japanese Pharmacopoeia-approved No. 30 test sieve (Tokyo Screen Co., Ltd., Tokyo, Japan). Ultrafine crystalline lactose (approximately 435.0 g) was added to the sieved powder to make 500 g of 10 mg/g baclofen powder, which was mixed by an automatic mixer (YM-500, Yuyama Corporation, Tokyo, Japan) at 620 rpm for 60 s at 20 rpm.

### 2.3. Drug Stability Study

#### 2.3.1. Experimental Design

The compounded baclofen powder was stored in the constant humidity/temperature chamber (Yamato Scientific Co., Ltd., Tokyo, Japan) at 25 ± 2 °C/60 ± 5% RH. [19]. The stability was assessed on samples withdrawn according to three schedules: (1) a ‘bottle (closed)’ study during which samples were withdrawn from distinct polycarbonate amber bottles (500 g of compounded baclofen powder kept in the bottle with desiccant) on day 0 and after 30, 60, 90, and 120 days of storage, (2) a ‘bottle (in use)’ study [20], where successive analyzes were performed on samples withdrawn from the same amber polycarbonate bottle (500 g of compounded baclofen powder kept in the bottle with desiccant) for powders (Yamayu Co., Ltd., Osaka, Japan), after daily removal of 0.1 g in the clinical setting during storage periods, (3) a ‘laminated’ study during which samples were withdrawn from a cellophane and polyethylene-laminated paper package (0.3 g of compounded baclofen powder in each package) (TK70W, Takazono Co., Ltd., Tokyo, Japan). The contents of baclofen have to comply with the specifications of 90.0–110.0% of the initial contents [21]. Therefore, the percentage of drug contents was calculated as (measured concentration/initial concentration) ×100 (%).

#### 2.3.2. Instrumentation and Chromatographic Conditions

For the detection of baclofen and its degradants, a previously validated liquid chromatography-diode array detection (LC-DAD) method was used [17,22]. The LC system was the ‘Ultimate 3000 HPLC System’ (Thermo Fisher Scientific K., Tokyo, Japan), consisting of an autosampler, column oven, and DAD. The autosampler was set at 10 °C and the column oven at 40 °C. Chromatographic separation was performed using a C18 column (Imtakt US-C18 column; length150 mm, inner diameter 3.0 mm, particle size 5 µm; Imtakt Co., Ltd., Kyoto, Japan). Eluent A was 10 mM ammonium formate solution, and eluent B was 0.1% acetonitrile formate solution. Separations were made by isocratic mode with 60% B composition at a constant flow rate of 0.4 mL/min. The separation time was 8 min. The eluent was filtered through a 0.22 µm filter (Merck Millipore, Darmstadt, Germany). The injection volume was 5 µL. Detection was carried out at 220 nm. Data recording and reprocessing were performed using Chromeleon software version 6.80 (Thermo Fischer Scientific K.K., Tokyo, Japan).

#### 2.3.3. Assay for Known Baclofen Impurities (A and B)

Using UV detection at 220 nm, the previously known baclofen impurities A and B were identified [17]. Their retention periods were collected in order to identify and quantify them during stability testing. By comparing the relative peak areas of baclofen, the contents of each impurity were assessed.

#### 2.3.4. Validation of Quantitative Analysis

The prepared mixed standard stock solution containing baclofen, baclofen impurity A, and baclofen impurity B was diluted to a series of appropriate concentrations to construct calibration curves.

##### Calibration Standards and Quality Control

Each calibration standard curve was constructed by preparing six samples ranging in quantity from 2.0 to 20.0 μg/mL (2.0, 4.0, 6.0, 8.0, 10.0, and 12.0 μg/mL) for baclofen, and 0.01 to 1.0 µg/mL (0.01, 0.05, 0.1, 0.2, 0.5, and 1.0 µg/mL). These samples were also used for quality control (QC) and were stored at − 80 °C. The acceptance criterion for linearity is that the correlation coefficient should not be less than 0.99.

##### Limit of Detection (LOD) and Lower Limit of Quantification (LLOQ)

The lower limit of detection (LOD) was determined based on the standard deviation of the response of the curve (Sy) and the slope of the calibration curve (S); LOD was determined by multiplying Sy/S by 3.3. The standard deviation of the response was determined based on the standard deviation of the *y*-intercept of the regression line. The lower limit of quantification (LLOQ) was set to Sy/S multiplied by 10; LOD and LLOQ were calculated for impurities A and B only.

##### Precision and Accuracy

Measurement of the six concentrations was repeated three times per day and repeated for three days. The obtained data were statistically analyzed by one-way analysis of variance, and the intra-day and inter-day accuracy and precision were calculated. The deviation of the mean from the true value was expected to be within 15% of the actual value.

### 2.4. Drug Release Study

The dissolution test was performed according to the Japanese Pharmacopoeia 17.6.10 (paddle method; NTR-6400AC; Toyama Sangyo, Tokyo) according to ICH by stirring 900 mL eluate at 37 ± 0.5 °C, 50 rpm. Purified water was used as the medium according to the test method of the original tablets [8]. At each sampling time, 1.5 mL of the eluate was collected, filtered (0.22 μm syringe filter; Millipore, Darmstadt, Germany), and stored in test vials at −20 °C until analysis. The compounded and stored powders were transferred to a dissolution vessel with wax paper and tested for dissolution. Six samples each were taken from the dissolution tester and analyzed by the LC-DAD method. The average elution rate of each sample was compared to the sample formulated on day 0. The similarity of dissolution profiles was evaluated using the f2 statistic defined in the Ministry of Health, Labor, and Welfare guidelines [23].

### 2.5. Differential Scanning Calorimetry (DSC)

Differential Scanning Calorimetry (DSC) was utilized to investigate the physicochemical compatibilities and solid interaction of baclofen and lactose monohydrate after storage. The samples (4 mg) were heated (50 to 300 °C) at a constant scanning speed (10 °C/min) in sealed aluminum pans, using nitrogen as purging gas (25 mL/min) using DSC-60 Plus (Shimadzu Co., Ltd., Kyoto, Japan). Only the ‘bottle (closed)’ samples were subjected to DSC analysis.

### 2.6. Powder X-ray Diffraction Analysis

Stored samples in ‘bottle (closed)’ storage condition after storing for 60, 90, and 120 days were analyzed via powder X-ray diffraction (PXRD). The PXRD analysis was carried out on a RINT 2000 (Rigaku Co., Tokyo, Japan). Measurements of the crystallinity of the obtained solid phase were performed at 40 kV voltage, 40 mA current, a scanning speed of 4°/min, Ni filter, and a radiation source of CuKα_1_.

### 2.7. Color Evaluation

The color was determined by the CR-400 color difference meter (produced by Konica Minolta Co., Ltd.; Tokyo, Japan). Prior to collecting the samples, the bottles were vertically shaken 10 times, and 1.0 g of sample was weighed and poured into the quarts cell for fluorescence study. The color of the samples was determined in triplicate. The color difference ΔE*_ab_ was defined and calculated as the change in Hunter’s parameters.

## 3. Results

### 3.1. Liquid Chromatography Method and Validation

**Figure 1:**
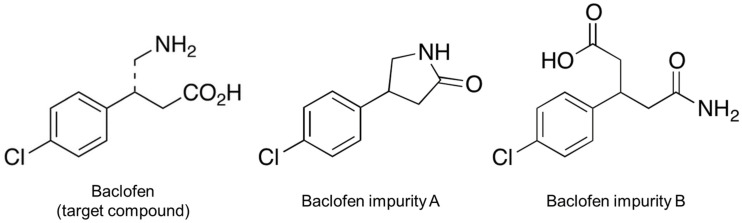
Structure of baclofen and its impurities.

The retention time of baclofen and baclofen impurities A and B were 1.9, 6.3, and 4.4 min, respectively (Figure 1). The chromatograms showed no interfering peak eluting at the retention times of baclofen and its impurities. The six-point calibration curve was found to be linear over the concentration range of 2.0–12.0 μg/mL for baclofen, and 0.01–1.0 µg/mL for baclofen impurities, respectively. The LOD and LLOQ estimated from the residuals of the regression standard deviations and the slope of the calibration curve for both impurities A and B were 0.005 and 0.01 μg/mL, respectively. The mean correlation coefficients for the calibrations were ≥0.99 for baclofen and its impurities, respectively. Recoveries ranged from 96.3% to 98.4%. The intra-day and inter-day precisions for the three compounds were less than 10% each, and the intra-day and inter-day accuracy at each six concentration levels ranged from 95.0% to 105.0% for all three compounds, which met the acceptance criteria. Recoveries ranged from 96.3% to 98.4%.

### 3.2. Stability Study

**Figure 2:**
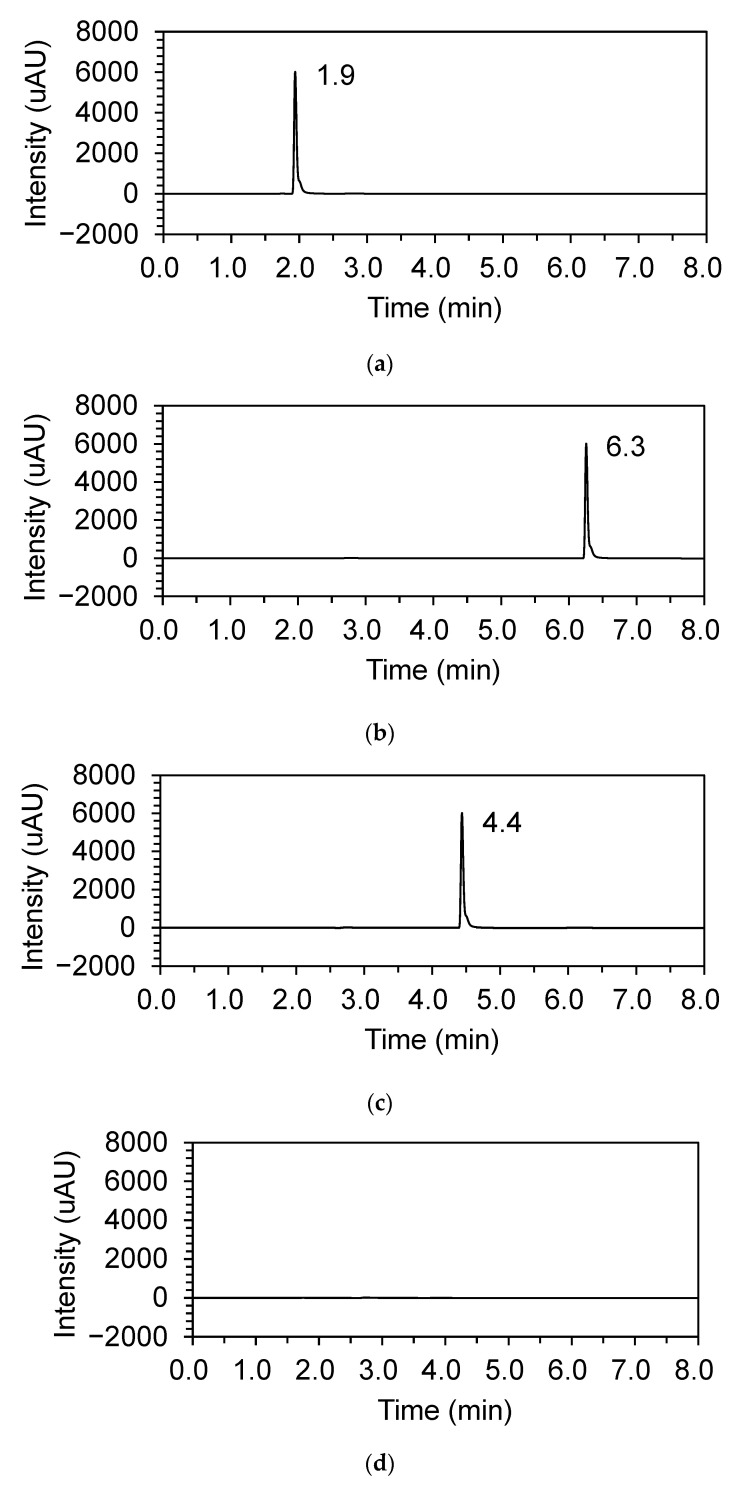
Chromatograms of 10 µg/mL of baclofen (**a**), impurity A (**b**), impurity B (**c**), and the solvent only (**d**).

The results of the stability study are presented in Table 1. Upon storage at 25 ± 2 °C/60 ± 5%RH, the baclofen content remained within the specifications of 90.0–110.0% of the initial concentration during 120 days in each ‘bottle (closed)’, ‘bottle (in use)’, and ‘laminated’ storage condition, respectively. Examples of chromatograms obtained for the analysis of baclofen of compounded products on day 0 and day 120 at 25 ± 2 °C/60 ± 5% RH in each sampling schedule are shown in Figure 2. No degradation of baclofen was observed during the investigation periods in each sampling schedule.

### 3.3. Impurity Study

**Figure 3:**
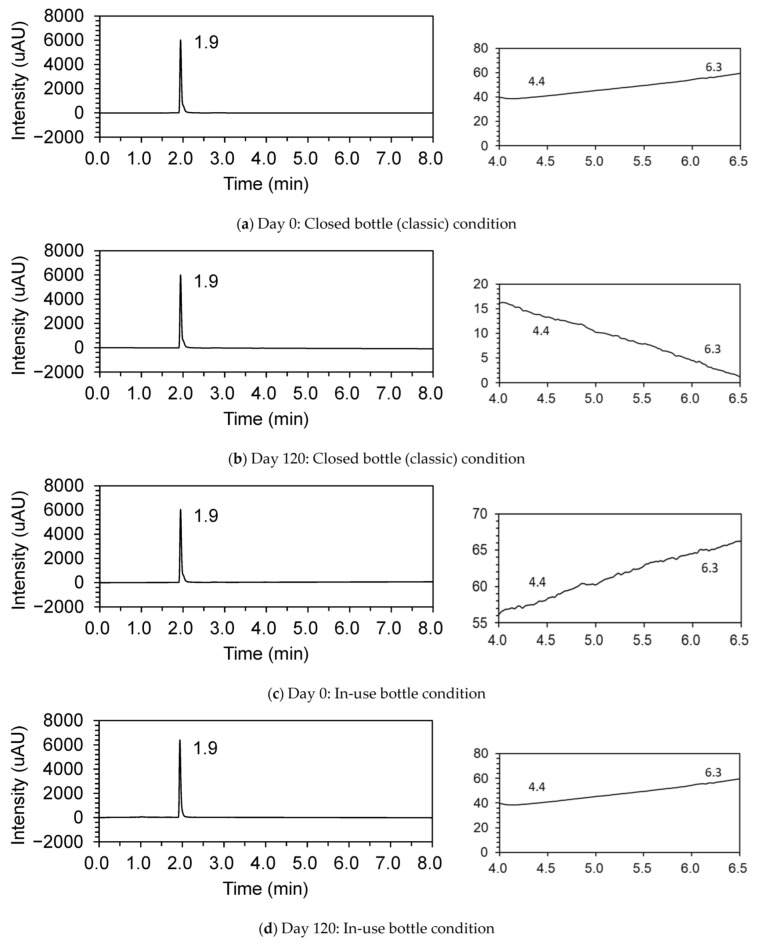

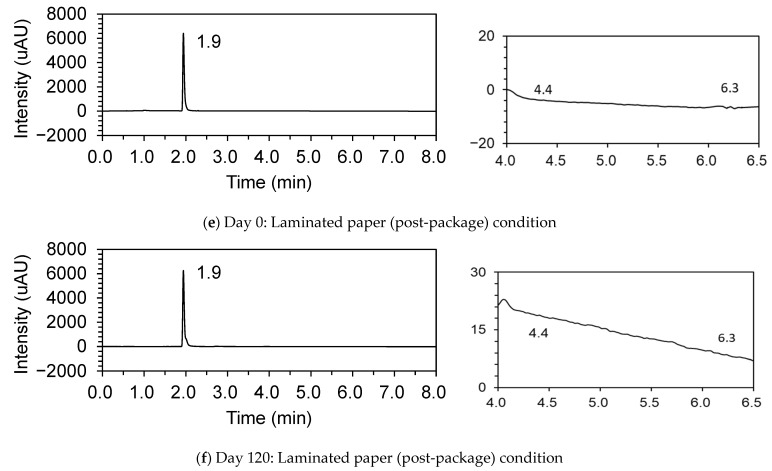
Dissolution profiles of compounded baclofen formula in water on days 0 and 120 in each ‘bottle (closed)’ (**a**,**b**), ‘bottle (in use)’ (**c**,**d**), and ‘laminated’ (**e**,**f**) condition. Enlarged chromatograms for confirmation of impurities A and B are shown to the right of each chromatogram. Each sample was examined with six replicates.

A reference chromatogram at 220 nm showed no impurities in the compounded powder (Figure 3).

### 3.4. Dissolution Test

**Figure 4:**
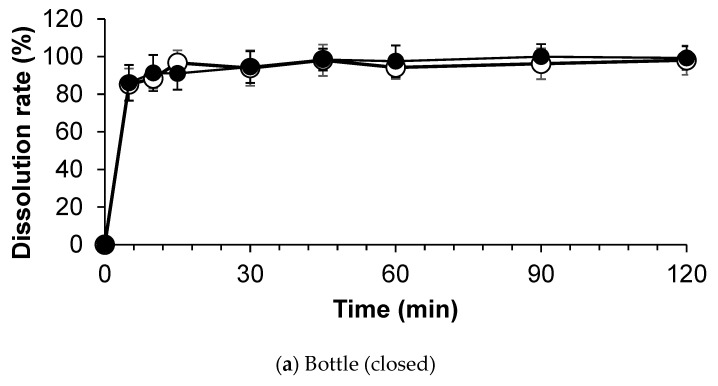

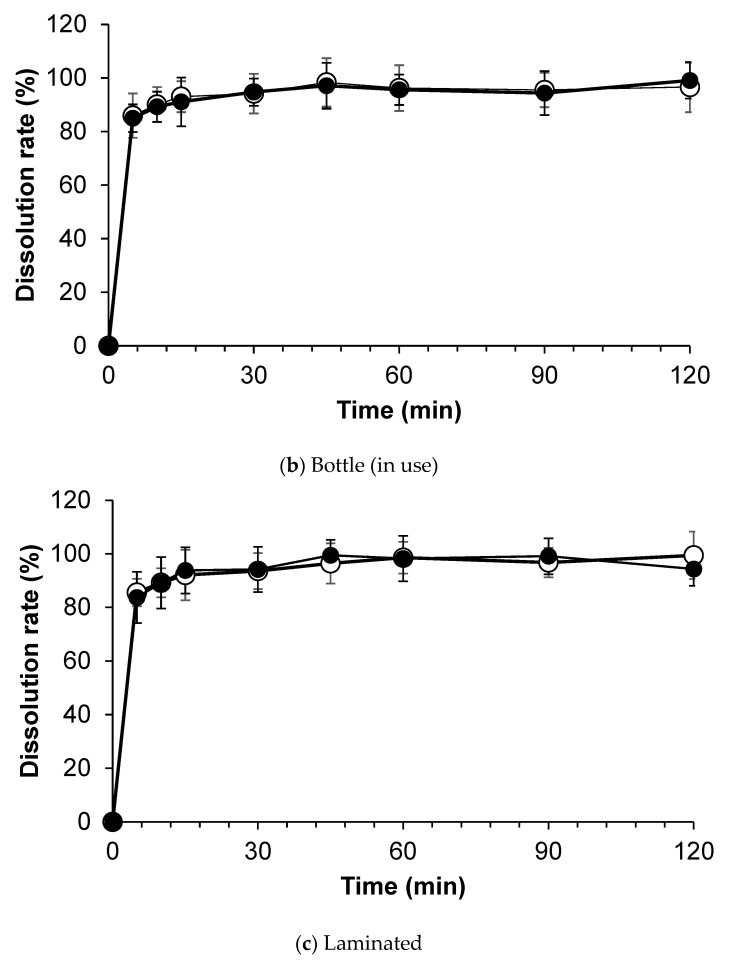
Dissolution profiles of compounded baclofen formula in water on days 0 (closed circles) and 120 (opened circles) in each condition. Each sample was examined in six replicates.

In dissolution studies in water, baclofen showed rapid and complete dissolution, all within 15 min. The dissolution profiles showed no significant variation in the stored combination powder compared to the crushed tablets on day 0 (Figure 4).

### 3.5. The Thermal Behavior of the Various Samples

**Figure 5:**
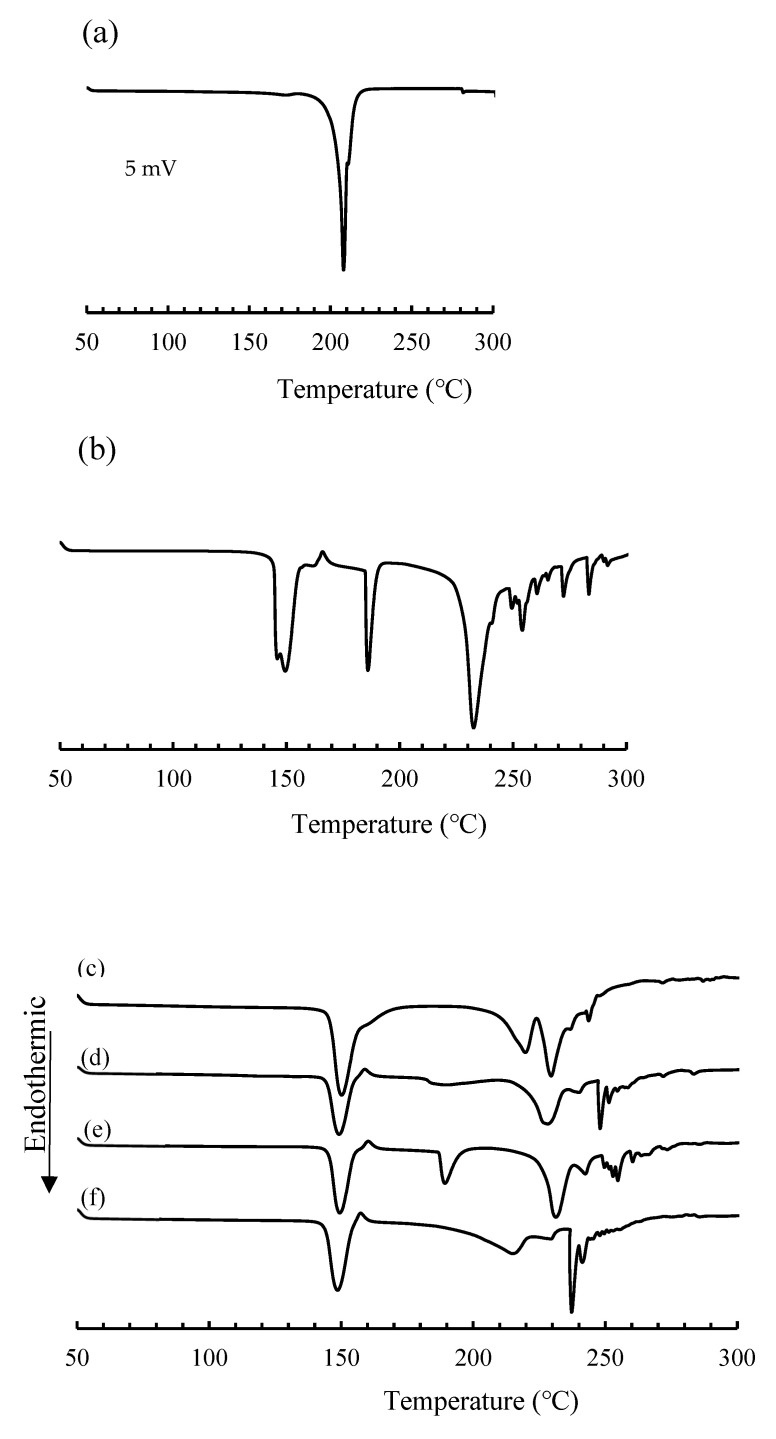
DSC curves of various samples. (**a**) Baclofen crystals, (**b**) lactose, (**c**) baclofen powder just after the preparation, (**d**) baclofen stored in the closed-bottle condition for 60 days, (**e**) 90 days, and (**f**) 120 days.

The thermal behavior of the various samples, baclofen crystals, lactose monohydrate, compound just after the preparation, and compound stored in the closed bottle condition for 60, 90, and 120 days are shown in Figure 5. One endothermic peak was observed in the DSC curve of baclofen crystals at one near 183 °C due to the fusion of crystals (Figure 5a). In contrast, some endothermic peaks correspond to the dehydration of bound water at 161 °C, a small exothermic peak due to the crystalline transition at 161 °C, and a fusion at 179 °C, followed by the beginning of decomposition (oxidation) at 211 °C observed in the DSC curve of lactose monohydrate. These thermal behaviors are consistent with what is reported in [24,25].

As for the curves of the compounds stored in the closed bottle condition for 60, 90, and 120 days, the endothermic peak due to the fusion of baclofen crystals disappeared or shifted around 180–220 °C. Whereas, on the DSC curves for samples stored for 90 and 120 days (Figure 5e,f), endothermic peaks were observed at 190 °C and around 190–200 °C. Although this study could not clarify the cause of these peaks, Alzoubi et al. demonstrated that the solid-state epimerization from a-lactose to b-lactose is triggered by dehydration, and the change in the crystalline form of baclofen was suggested during the storage [26,27]; details of these thermal behaviors will be explained by using DSC-PXRD in a future study.

### 3.6. PXRD Analysis

**Figure 6:**
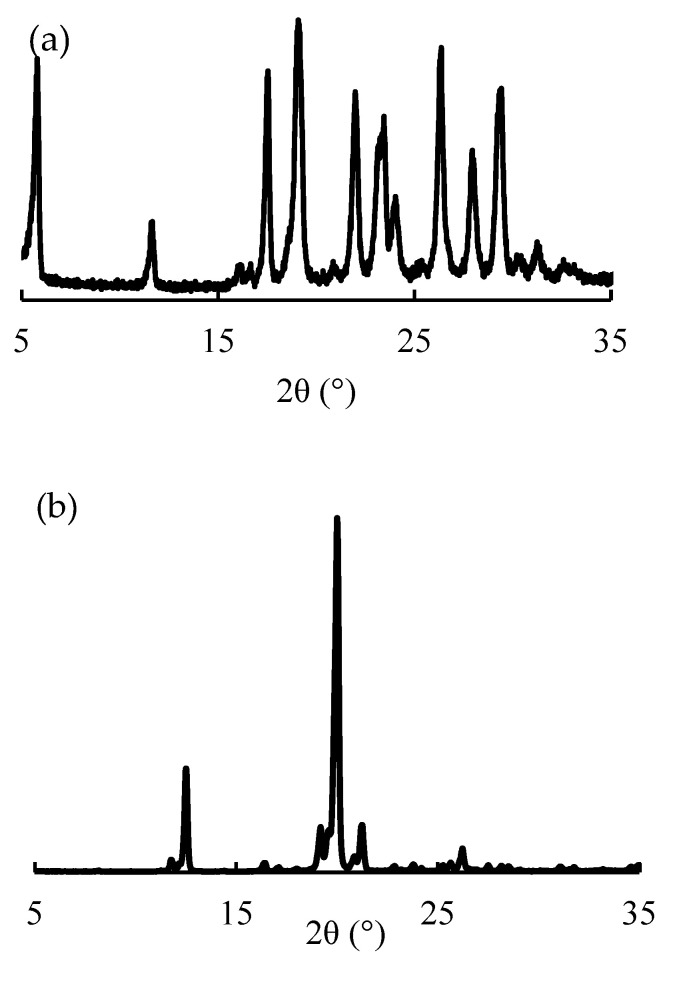

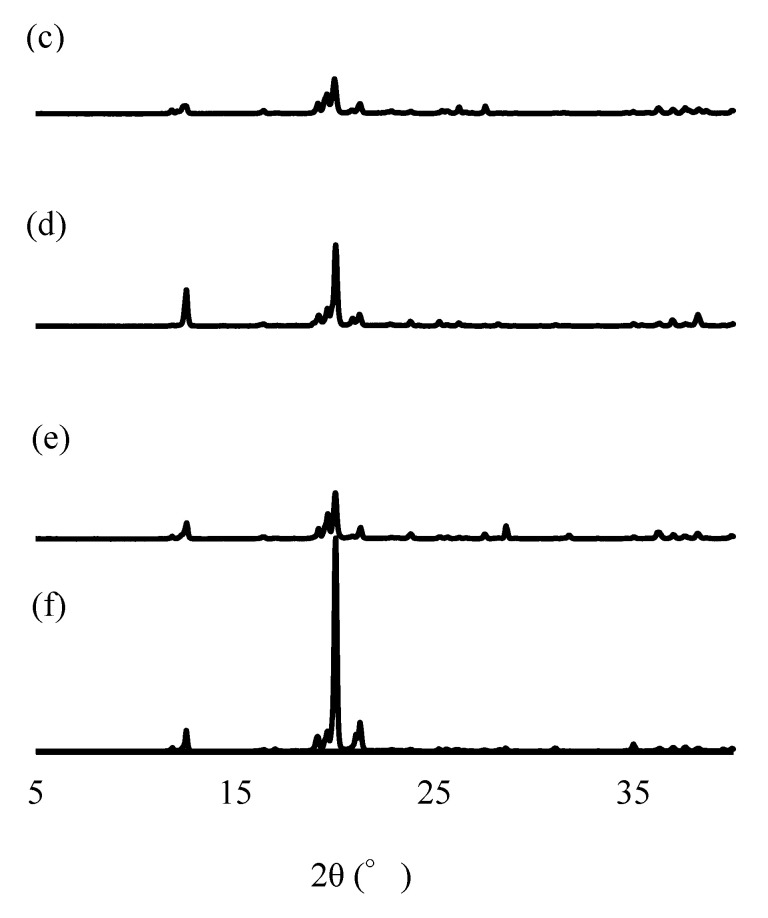
Powder X-ray diffraction patterns of various samples. (**a**) Baclofen crystals, (**b**) lactose, (**c**) baclofen powder just after the preparation, (**d**) baclofen stored in the closed-bottle condition for 60 days, (**e**) 90 days, and (**f**) 120 days.

The baclofen crystals showed characteristic peaks at 2θ = 17.5, 19.1, 22.0, 23.4, 26.4, 28.0, and 29.4°, representing their crystalline nature (Figure 6a). In contrast, the diffraction pattern of lactose monohydrate shows characteristic peaks at 2θ = 12.6, 19.2, 20.0, and 21.3° (Figure 6b). The peaks due to baclofen crystals disappeared in compounded powders because the amount of baclofen in the mixture was small compared to the amount of lactose; as for the diffraction peaks due to lactose monohydrate stored in the ‘bottle (closed)’ condition for 60, 90, and 120 days, no crystallographic changes between storage types were shown (Figure 6c–f). The angles at which diffraction peaks were observed for each sample were the same, but differences in peak intensity were observed. The difference in peak intensities is affected by the filling of the sample to the measurement plate, but the difference in peak intensities in this experimental system is due to the difference in the amount of moisture adsorbed on the sample. In addition to the results obtained from the DSC measurement, it is necessary to investigate the effect of water adsorption on the sample in detail in the future.

### 3.7. Color Evaluation

The color differences of compounds stored at various conditions are listed in Table 2. Although there are no significant differences between samples, color change during the storage did not occur in this study.

## 4. Discussion

Due to the fact that many commercially available oral formulations are not appropriate for pediatric usage and adult formulations are reformulated in the clinical setting by pharmacists and caregivers [14], off-label or unauthorized use of adult drugs often occurs. The development of pediatric formulations is supported by several stakeholders such as the World Health Organization [28], the European Medicines Agency, the U.S. Food and Drug Administration [29], Health Canada [30], and the European Pediatric Formulation Initiative (EUPFI) consortium [31] for better and safer medicines for pediatric patients, and Batchelor and colleagues review these challenges and current progress [32]. To improve the safety and compliance of enterally administered products, there is an urgent need to standardize and ensure the quality of oral dosage forms. However, at present, most compounding is conducted for liquid dosage forms. [33,34], and there are limited reports on the stability and safety of solid oral formulations. Concerning the stability of baclofen liquid formulation, Polonini and their colleagues demonstrated 90 days of stability of a 2.0 mg/mL extemporaneous aqueous baclofen solution compounded with SyrSpend SF PH4 at room temperature [35]. Johnson and their colleagues also demonstrated 35 days’ stability of baclofen in tablet-dissolved solutions in simple syrup NF, which were kept at 4 °C in amber glass [36]. A 10 mg/mL baclofen suspension by crushing baclofen tablets prepared in a 1:1 mixture of Ora-Sweet and Ora-Plus (Paddock Laboratories), a 1:1 mixture of Ora-Sweet SF and Ora-Plus (Paddock Laboratories), and cherry syrup stored at 25 °C in amber, clear polyethylene terephthalate bottles also maintained stability for up to 60 days [37]. We report the first stability study of a 10.0 mg/g baclofen oral powder prepared from commercially available baclofen tablets. Stability studies showed that the baclofen content was unchanged, and baclofen-related impurities (baclofen impurities A and B) were not detected in the LC-DAD method. The results also indicated that there was no chemical desorption or adsorption of baclofen on the polycarbonate container and packaging. In the present study, the thermal behavior and powder X-ray diffraction of the compounded baclofen were analyzed, but the only conditions under which they were performed were ‘closed bottle’ conditions. Although this condition is the most typical storage environment for powder in an actual dispensing setting, it is necessary to study the changes that occur when the powder is dispensed in divided packages or opened bottle conditions.

Additionally, since this study did not include information on the efficacy of baclofen or the side effects on patients, we believe that further information on the efficacy and safety of baclofen in modified dosage forms is essential. In the EU and the USA, pharmaceutical companies are increasingly required to present age-appropriate development strategies for new drug development [29,38,39]. In addition, in other countries, attempts are being made to standardize dosage form changes [40,41,42,43]. A similar approach may be required to standardize drug compounding for pediatric patients in Japan.

In this study, we conducted baclofen powder’s stability and physicochemical tests prepared from commercially available tablets. As a result, baclofen in compounded powder maintained its quantity for at least 120 days in each ‘bottle (closed),’ ‘bottle (in use)’, and ‘laminated’ storage conditions. It is hoped that the established formulation method will guarantee the quality of the pediatric baclofen formulation and contribute to the standardization of baclofen formulations in various pharmacies and medical facilities in Japan.

## 5. Conclusions

Baclofen powder prepared from commercially available tablets is stable at 25 ± 2 °C/60 ± 5% RH for 120 days in unopened, daily opening, and laminated conditions. We expect that this standardization of dosage form changes will help ensure the safety of pediatric drug therapy by providing homogeneous and quality-assured dosage form changes at each pharmacy.

## Figures and Tables

**Table 1:** Compounded baclofen stability.

Study Methods	Storage Conditions	Storage Container	Test Periods (Days)				
			0	30	60	90	120
			Baclofen Concentrations *				
Bottle (closed)	25 °C ± 2 °C/60% ± 5% relative humidity	Amber/PC bottle	100.0%	100.2 ± 3.2%	99.8 ± 2.8%	99.0 ± 1.6%	99.6 ± 2.7%
Bottle (in use)		Amber/PC bottle	100.0%	99.9 ± 2.9%	98.9 ± 4.1%	99.1 ± 2.5%	98.7 ± 3.1%
Laminated paper		Amber/CP laminated paper	100.0%	101.0 ± 2.2%	99.7 ± 1.3%	100.3 ± 2.7%	99.6 ± 1.2%

PC, polycarbonate; CP, cellophane, and polyethylene. * Baclofen concentrations are presented as percentages of the day 0 content (100%).

**Table 2:** Color evaluation of various samples.

Storage Periods	Color Difference (ΔE_ab_)
0 days	16.75 ± 0.102
60 days	16.72 ± 0.208
90 days	18.26 ± 0.046
120 days	16.29 ± 0.128

Data are expressed as mean ± S.D. (*n* = 3).

## Data Availability

Not applicable.
